# Multiple Clay Shoveler’s Fractures of the Thoracic Spine

**DOI:** 10.3390/diagnostics12092190

**Published:** 2022-09-09

**Authors:** Wilbur Teng Jun Hoong, Arun-Kumar Kaliya-Perumal

**Affiliations:** 1 Lee Kong Chian School of Medicine, Nanyang Technological University, Singapore 636921, Singapore; 2 Melmaruvathur Adhiparasakthi Institute of Medical Sciences and Research, Melmaruvathur 603319, Tamil Nadu, India

**Keywords:** conservative treatment, fractures, occupational injuries, spine, spinous processes

## Abstract

Typically, a clay shoveler’s fracture is a stress-type avulsion fracture involving the spinous processes of the lower cervical or upper thoracic vertebrae. Even though C7 and T1 are the most commonly involved spinal levels, these avulsion fractures can occur at any lower cervical or upper thoracic level, either as solitary or multiple fractures. This fracture used to be common in workers who shovel heavy loads of clay for long periods, hence its name. It does not cause any structural, functional, or neurological impairments and is therefore considered a stable fracture. Management is mostly conservative, involving rest, analgesics, and activity modification for a period of 4–6 weeks. Here, we present a 35-year-old male who sustained a motor vehicle accident. Except for midline tenderness in the back, there were no other positive findings. Plain radiographs showed a T11 vertebral compression fracture and absent or deviated spinous process shadows for most of the upper thoracic vertebrae. Computed tomography (CT) imaging clearly revealed multiple spinous process fractures extending from T2 to T8 levels. Considering the stability of these fractures, the patient was managed conservatively with rest, bracing, and analgesics. The recovery was quick, and he was back to his full functional status by six weeks.

##  

**Figure 1:**
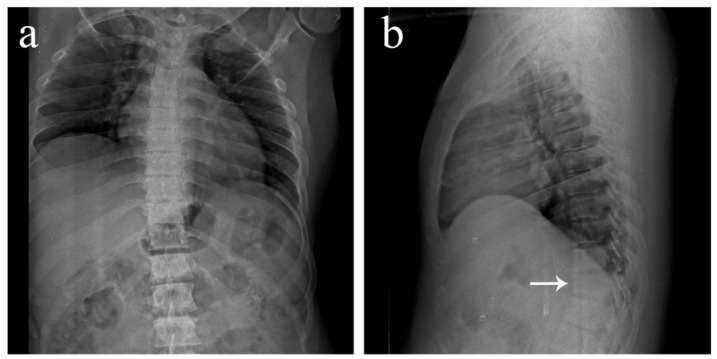

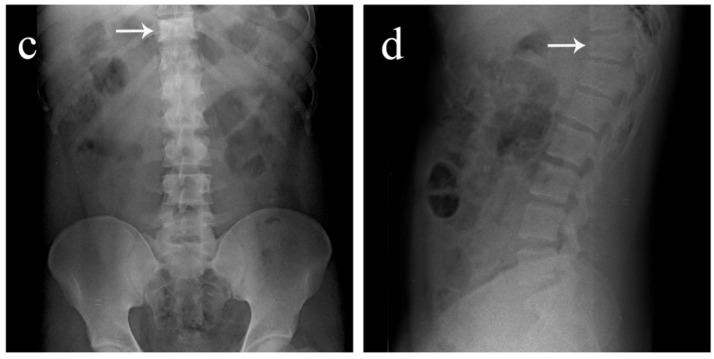
Antero-posterior and lateral view plain radiographs of a 35-year-old male who was brought to the emergency department with multiple abrasions over the trunk and pain in the upper back following a motor vehicle accident. He was conscious and well oriented with stable vitals. Upon examination, midline tenderness was elicited throughout his upper back. His motor power was 5/5 in all four limbs, and there was no sensory deficit. Plain radiographs revealed an obvious anterior wedge compression fracture of the T11 thoracic vertebra. However, since tenderness was elicited throughout the upper thoracic spine and not confined just to the T11 level, more fractures were suspected. On further examination of the anteroposterior view radiograph, it was noticed that the spinous process shadows of the upper thoracic vertebra were either absent or deviated. This brought suspicion of multiple spinous process fractures, which were later confirmed using a computed tomography (CT) scan. (**a**) Antero-posterior view of the thoracic spine showing absent or deviated spinous process shadows throughout the upper thoracic spine, raising suspicion of spinous process fractures. (**b**) Lateral view of the thoracic spine showing a compression fracture of T11 vertebrae (arrow). (**c**) Antero-posterior view of the lumbar spine where the T11 vertebra appears to be sclerotic (arrow). (**d**) Lateral view of the lumbar spine showing normal lordosis and the T11 compression fracture (arrow).

**Figure 2:**
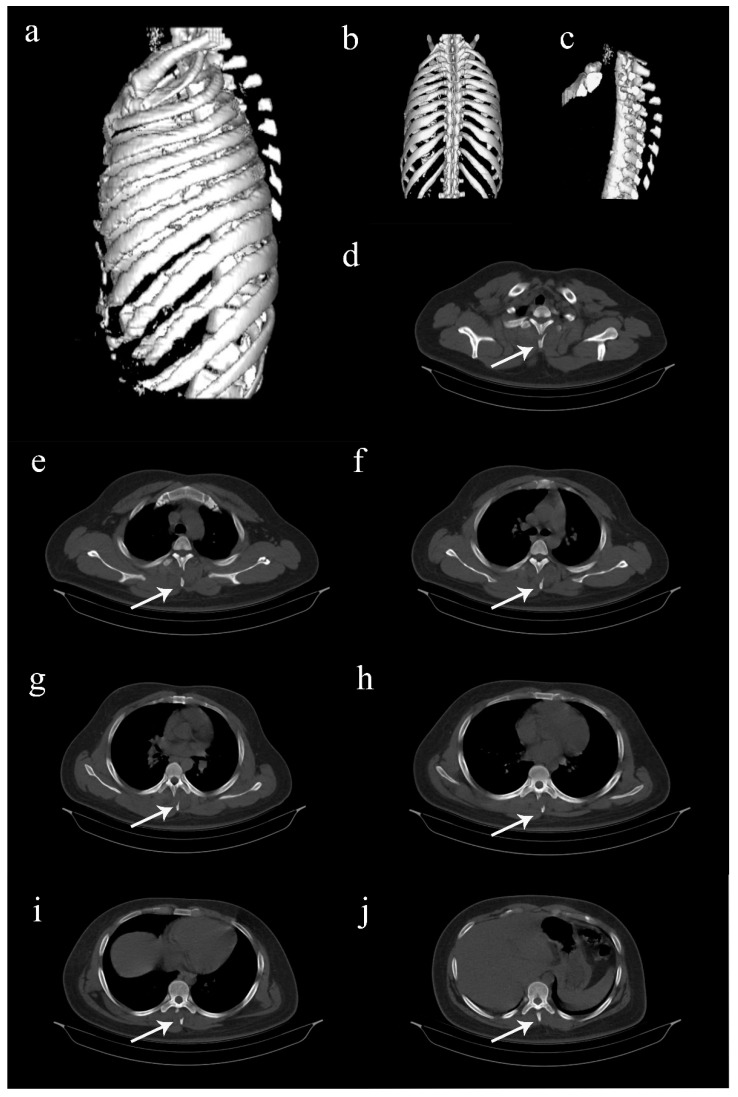
Computed tomography (CT) images of the thoracolumbar spine. (**a**–**c**) Three-dimentional reconstructed CT images showing multiple spinous process fractures extending from T2 to T8 levels, resembling clay shoveler’s fractures [1,2,3,4,5]. (**d**–**j**) Axial view CT images extending from T2 to T8 levels, showing the fractured spinous processes (arrows). Such involvement of multiple spinous processes in the thoracic spine is rare, and hence, sparsely reported [1,2,3]. Considering the stability of these fractures, it was decided to treat the patient conservatively. He was put on a thoracolumbar corset and was advised adequate bed rest for two weeks. Analgesics were provided, and self-care for the abrasions was taught. Protected weight bearing and mobilization exercises were started on the second week while on thoracolumbar corset support. The recovery was quick, and the patient was back to his full functional status by 6 weeks. Further follow-up was uneventful.

## Data Availability

Not applicable.

## References

[1] Kazanci A., Gurcan O., Gurcay A.G., Turkoglu O.F., Bavbek M. (2015). Six-level isolated spinous process fracture of the thoracic vertebrae (clay-shovelerߣs fracture) and a review of the literature. Neurol. India.

[2] Han S.R., Sohn M.J. (2014). Twelve contiguous spinous process fracture of cervico-thoracic spine. Korean J. Spine.

[3] Akhaddar A., Mandour C. (2013). Multiple contiguous cervicothoracic Clay-shoveler’s fractures (from C6 to T9 spinal vertebrae). Pan Afr. Med. J..

[4] Toman E., Beaven A., Harland S., Porter K. (2016). Clay-shoveler’s fracture: A snapshot. Trauma.

[5] Posthuma de Boer J., van Wulfften Palthe A.F., Stadhouder A., Bloemers F.W. (2016). The Clay Shoveler’s Fracture: A Case Report and Review of the Literature. J. Emerg. Med..

